# A Comprehensive Review on Unveiling the Journey of Digoxin: Past, Present, and Future Perspectives

**DOI:** 10.7759/cureus.56755

**Published:** 2024-03-23

**Authors:** Rahul Khandelwal, Jayant D Vagha, Revat J Meshram, Ankita Patel

**Affiliations:** 1 Pediatrics, Jawaharlal Nehru Medical College, Datta Meghe Institute of Higher Education and Research, Wardha, IND

**Keywords:** clinical applications, pharmacology, atrial fibrillation, heart failure, cardiac glycoside, digoxin

## Abstract

Digoxin, a cardiac glycoside derived from the foxglove plant (*Digitalis* spp.), has been utilized for centuries in managing various cardiac conditions due to its ability to increase myocardial contractility and regulate heart rate. This comprehensive review explores the historical context, pharmacological properties, clinical applications, efficacy, safety profile, challenges, and future perspectives of digoxin. Tracing its journey from traditional medicine to modern cardiovascular therapeutics, we delve into its mechanism of action, therapeutic indications, and clinical guidelines. While digoxin remains a cornerstone therapy for heart failure and atrial fibrillation, its narrow therapeutic index and individual variability in response pose challenges in clinical practice. Nevertheless, ongoing research efforts aim to elucidate its role in emerging therapeutic areas and technological advancements in drug delivery. Despite the advent of newer pharmacological agents, digoxin's enduring relevance lies in its established efficacy, affordability, and global accessibility. This review underscores the symbiotic relationship between tradition and progress in cardiovascular medicine, highlighting the timeless pursuit of medical innovation to optimize patient care.

## Introduction and background

Digoxin, a cardiac glycoside derived from the foxglove plant (*Digitalis* spp.), has been a cornerstone in managing various cardiac conditions for centuries. Its ability to increase myocardial contractility and regulate heart rate has made it a mainstay in treating heart failure, atrial fibrillation, and other related conditions [1]. To truly appreciate the significance of digoxin in contemporary medicine, it is essential to delve into its rich historical background. The journey of digoxin from its discovery in traditional medicine to its current clinical applications reflects the evolution of pharmacology and the enduring quest for effective cardiac therapeutics [2].

This review aims to provide a comprehensive overview of digoxin, encompassing its historical origins, pharmacological properties, clinical applications, efficacy, safety profile, challenges, and future perspectives. By synthesizing past, present, and future perspectives, this review seeks to enhance understanding of digoxin's role in modern cardiovascular medicine and shed light on potential future research and clinical practice directions.

## Review

Historical background

Discovery and Early Use of Digitalis Plants

Early mentions and erratic use: *Digitalis*, derived from the foxglove plant, garnered early mentions in medical writings as far back as 1250 by the Welsh family of physicians known as the Physicians of Myddvai, who included it in their prescriptions [3]. However, its utilization remained sporadic until the 18th century. During this period, William Withering, an esteemed English physician and botanist, significantly contributed to its understanding and medical application. Withering's seminal monograph, published in 1785, meticulously described the clinical effects of an extract of the foxglove plant, thus popularizing its medical use [4,5].

William Withering's contributions: In his groundbreaking work titled "An Account of the Foxglove," Withering not only elucidated the indications for digitalis but also highlighted its potential toxicity [4,5]. His meticulous observations led to recommendations regarding the optimal timing for gathering foxglove leaves for medicinal purposes. Withering's advocacy for digitalis extended to various ailments, including dropsy and epilepsy, marking a significant advancement in medical practice [4,5].

Chemical composition and mechanism: Digoxin, a prominent cardiac glycoside derived from digitalis, is therapeutic by inhibiting the Na+/K+ ATPase pump in cardiac muscle cells [4,5]. This inhibition leads to an accumulation of intracellular calcium, thereby enhancing the efficiency of cardiac muscle contraction. This mechanism underpins digoxin's efficacy in managing conditions such as congestive heart failure and atrial arrhythmias [6].

Toxicity and visual effects: Despite its therapeutic benefits, digitalis possesses a narrow therapeutic window and can lead to toxicity in excessive doses. Symptoms of digitalis toxicity include vomiting, disturbed vision (xanthopsia), bradycardia, convulsions, unconsciousness, and potentially fatal outcomes [4,5]. Notably, the visual disturbances associated with digitalis toxicity, including a yellow tint to vision, have been linked to the works of the renowned artist Vincent van Gogh during his "yellow period," providing a striking example of the intersection between medicine and art [4].

Isolation and Purification of Digoxin

Digoxin, a cardiac glycoside employed in treating heart conditions, was initially isolated in 1930 by Dr. Sydney Smith from the foxglove plant *Digitalis lanata* [6]. The isolation process entailed extracting the glycosides from the woolly foxglove, yielding digoxin as one of the resultant compounds [6]. Subsequently, the purification of digoxin involved breaking down its glycosidic bonds within the body, forming digitoxin and sugars [6]. The historical significance of digoxin's discovery lies in its herbal origins rather than laboratory synthesis. In 1775, English physician William Withering discovered the therapeutic potential of the foxglove plant in treating conditions such as dropsy (edema) [5,7]. This breakthrough led to the widespread use of digitalis for medical purposes. Digoxin continues to be derived from the foxglove plant through a modern manufacturing process, which involves extracting digitalis from dried foxglove leaves to produce pure digoxin for pharmaceutical applications [5,7]. The purification and extraction of digoxin from *Digitalis lanata* have facilitated its extensive utilization in managing various cardiac conditions over the past century. This historical journey underscores the enduring importance of natural sources in drug discovery and development.

Early Clinical Applications and Controversies

Digoxin, a treatment for heart failure, has been in use for over two centuries since its introduction by William Withering in the late 18th century [8,9]. Derived from the *Digitalis purpurea* plant, commonly known as foxglove, digoxin was initially employed to manage cardiac dropsy, now recognized as congestive heart failure [9]. Despite its historical roots, the FDA only approved digoxin for heart failure treatment in the late 1990s [10]. In recent years, its usage has declined due to the emergence of newer therapies demonstrating efficacy in reducing mortality rates in heart failure [9]. However, digoxin remains integral in heart failure management, especially in cases involving systolic dysfunction and normal sinus rhythm [10,11]. Its application has extended to conditions such as atrial fibrillation and post-myocardial infarction management [10]. Nevertheless, recent studies have yielded mixed results regarding digoxin's impact on morbidity and mortality, sparking controversy surrounding its use [10,12]. Despite these challenges, digoxin continues to be recommended among select heart failure patients in the latest guidelines, underscoring its enduring role in cardiovascular care [11,12].

Pharmacology of digoxin

Mechanism of Action

Digoxin exerts its primary mechanism of action by inhibiting the sodium-potassium adenosine triphosphatase (Na+/K+ ATPase) enzyme, particularly within the myocardium. This inhibition results in an accumulation of intracellular sodium, which diminishes the sodium-calcium exchanger's activity. Consequently, intracellular calcium levels elevate, facilitating increased calcium binding to troponin C and augmenting myocardial contractility [13]. Furthermore, digoxin functions as a negative chronotropic agent, effectively reducing heart rate [6]. Given its narrow therapeutic window, meticulous monitoring of serum digoxin levels is imperative to mitigate the risk of potential toxicity.

Pharmacokinetics

Digoxin, a cardiac glycoside medication, is widely used to treat various heart conditions, including congestive heart failure, atrial fibrillation, and certain cardiac arrhythmias [6]. Its primary mechanism of action revolves around inhibiting the sodium-potassium adenosine triphosphatase (Na+/K+ ATPase), predominantly within the myocardium. This inhibition enhances myocardial contractility, stroke volume, and blood pressure while concurrently reducing heart rate [14]. However, digoxin's therapeutic range is narrow, and its toxicity can manifest through symptoms such as nausea, vomiting, visual changes, and arrhythmias [6]. Digoxin is incompletely absorbed when administered orally, with a significant portion cleared by the kidneys, resulting in 70-85% excretion via urine [14]. The bioavailability of an oral dose varies from 50-90%, although gelatinized digoxin capsules have been reported to have a bioavailability of 100% [14-15]. Typical dosages range from 0.01-0.02 mg/kg for loading doses, followed by 0.125-0.25 mg/day for maintenance therapy [14]. Despite being utilized for over two centuries, digoxin's usage has declined with the emergence of novel therapies; nevertheless, it remains an integral component of standard care for heart failure [6]. Recent studies have yielded conflicting results regarding digoxin's impact on morbidity and mortality, necessitating further investigation and refinement of its future role based on emerging evidence [6].

Pharmacodynamics

About 70 to 80% of an oral digoxin dose is absorbed, mainly in the proximal part of the small intestine. The degree of binding to serum albumin is 20 to 30%. Digoxin is extensively distributed in the tissues, as reflected by the large volume of distribution [16]. The pharmacodynamic effects of digoxin are intricately linked to its uptake in the heart following administration. Toxic symptoms associated with digoxin correlate with its concentration in the heart and serum levels [15]. Given its narrow therapeutic window, factors, such as renal function, body weight, serum albumin concentration, and drug interactions, can significantly influence the pharmacokinetics and pharmacodynamics of digoxin [6,15]. Despite its status as one of the oldest medications in cardiology, digoxin remains a valuable treatment option for conditions such as atrial fibrillation and heart failure. However, its utilization necessitates vigilant monitoring due to the potential for toxicity, particularly in cases involving supratherapeutic doses or chronic overexposure [6]. The profound pharmacodynamic effects of digoxin on the cardiovascular system underscore its pivotal role in the management of various heart conditions.

Therapeutic Indications

Digoxin finds primary application in the treatment of heart conditions, including congestive heart failure, atrial fibrillation, and specific cardiac arrhythmias [17]. Typically, it is administered alongside other medications such as diuretics and angiotensin-converting enzyme (ACE) inhibitors [17]. However, due to its narrow therapeutic range, digoxin's toxicity can lead to symptoms such as nausea, vomiting, visual disturbances, and arrhythmias [13]. Despite the advent of newer therapeutic options, digoxin remains a standard component of heart failure management [11]. Notably, the American Heart Association (AHA), American College of Cardiology (ACC), and Heart Failure Society of America (HFSA) endorse digoxin as a recommended medication for select heart failure patients in their latest guidelines [18].

Clinical applications

Treatment of Heart Failure

Digoxin, classified as a cardiac glycoside, serves as a therapeutic option for mild to moderate heart failure and the management of ventricular response rates in chronic atrial fibrillation [6,19]. Its indications encompass the treatment of mild to moderate heart failure in adults, augmentation of myocardial contraction in pediatric patients with heart failure, and regulation of ventricular rate in adults with chronic atrial fibrillation [6]. Renowned for its positive inotropic and negative chronotropic effects, digoxin enhances the force of cardiac contractions while reducing heart rate, which is particularly beneficial in atrial fibrillation [6]. Despite the introduction of newer therapies that have demonstrated efficacy in reducing mortality rates in heart failure, recent trials suggest that digoxin may still confer therapeutic benefits when employed judiciously [6,9]. However, the 2022 AHA/ACC/HFSA Guideline refrains from recommending routine digoxin usage in heart failure with reduced ejection fraction due to the absence of evidence supporting a mortality benefit [20]. Nevertheless, digoxin has shown efficacy in reducing hospitalizations related to worsening heart failure [20], often administered in conjunction with a diuretic and an ACE inhibitor when clinically feasible [6].

Management of Atrial Fibrillation

Digoxin, a cardiac glycoside, is its indications encompass various conditions, including heart failure and the maintenance of ventricular rate control in adult patients with chronic atrial fibrillation [20]. Operating as a positive inotropic and negative chronotropic agent, digoxin enhances cardiac contractility while reducing heart rate, which is particularly advantageous in atrial fibrillation management [20]. Despite its longstanding history as a treatment for heart failure, recent trials have not definitively established its efficacy in reducing mortality rates [9,20]. Nevertheless, digoxin has demonstrated the potential to decrease hospitalizations attributable to worsening heart failure [12]. The decision to utilize digoxin for managing atrial fibrillation or heart failure should consider individual patient characteristics and potential risks [12,20]. Thus, a comprehensive evaluation of the patient's clinical status and therapeutic goals is paramount in determining the appropriateness of digoxin therapy.

Other Therapeutic Uses

Abortion: Early investigations suggested that digoxin might induce uterine contractions, raising interest in its potential as an abortifacient. However, its effectiveness and safety concerns prompted limited utilization in this context [13,20]. The ambiguous findings led to cautiousness in its application for pregnancy termination, with the medical community opting for alternative approaches with better-established efficacy and safety profiles.

Arrhythmias: While digoxin is a standard therapy for atrial fibrillation, its exploration for managing other arrhythmias, such as ventricular tachycardia and ventricular fibrillation, has been undertaken. Nevertheless, its role in treating these conditions remains restricted compared to more contemporary treatments [6,20]. The advent of novel antiarrhythmic medications and interventions has marginalized digoxin's utilization in arrhythmia management, relegating it primarily to cases of atrial fibrillation with rapid ventricular response.

Hypertension: Research has investigated digoxin's potential to lower blood pressure, albeit with uncertain long-term efficacy in hypertension management. Consequently, it is not advocated as a first-line treatment for hypertension due to inconclusive evidence regarding its sustained effectiveness and concerns regarding its safety profile [20]. The emergence of numerous antihypertensive agents with established efficacy and safety profiles has diminished the significance of digoxin in hypertension management.

Cardioprotection: Studies have explored the potential cardioprotective effects of digoxin during surgical procedures or following acute myocardial infarction. However, the overall impact of digoxin on morbidity and mortality in these settings remains inconclusive [20]. Despite initial optimism surrounding its cardioprotective properties, subsequent research has yet to establish digoxin's role in improving clinical outcomes in surgical or post-infarction scenarios.

Angina pectoris: Digoxin has been investigated for its potential to alleviate chest pain associated with coronary artery disease. However, its efficacy in relieving angina pectoris is uncertain and is not typically employed [20]. The need for robust evidence supporting its efficacy in managing angina, coupled with the availability of more effective therapeutic options, such as nitrates and beta-blockers, has relegated digoxin to a marginal role in managing this condition.

Clinical Guidelines and Recommendations

The 2022 AHA/ACC/HFSA Guideline for the Management of Heart Failure advises against the routine administration of digoxin in patients with heart failure with reduced ejection fraction (HFrEF) due to the absence of evidence supporting a mortality benefit [21]. However, digoxin has effectively reduced hospitalizations related to worsening heart failure [20]. While digoxin has been utilized in the treatment of atrial fibrillation, retrospective studies have yielded conflicting findings regarding its safety and associated risks [12]. The utilization of digoxin necessitates vigilant monitoring owing to its narrow therapeutic window and potential for toxicity, which may precipitate arrhythmias and malignant hyperkalemia [20]. Management of digoxin toxicity primarily revolves around supportive therapy involving intravenous hydration and electrolyte regulation [20].

Efficacy and safety profile

Efficacy in Heart Failure Management

Digoxin has long been utilized as a treatment for heart failure; however, its efficacy and safety profile have been scrutinized since the advent of newer therapies. While digoxin has shown effectiveness in reducing heart rate during acute heart failure episodes triggered by tachyarrhythmia [22], caution is warranted, particularly in elderly patients [23]. Recent trials have failed to establish a mortality benefit associated with digoxin compared to newer heart failure treatments [9]. Nonetheless, certain studies indicate a potential benefit in reducing hospitalizations due to worsening heart failure [12]. The 2022 AHA/ACC/HFSA Guideline for the Management of Heart Failure refrains from recommending the routine use of digoxin in patients with heart failure with reduced ejection fraction, citing insufficient evidence for a mortality benefit [21]. Further research is imperative to elucidate the contemporary role of digoxin in heart failure management.

Safety Concerns and Adverse Effects

Digoxin is associated with various adverse effects, encompassing nausea, vomiting, headache, dizziness, loss of appetite, and diarrhea [24]. Elevated serum digoxin concentrations of approximately 0.9 ng/mL have been correlated with heightened mortality rates [23]. Moreover, digoxin has the potential to induce cardiac arrhythmias, particularly in individuals with pre-existing conduction abnormalities [24]. Additional side effects may include visual disturbances, confusion, and delirium [24]. Digoxin's interactions with other medications, such as azole antifungals, macrolide antibiotics, and St. John's wort, can impact its elimination from the body and heighten the risk of adverse reactions [24]. Hence, meticulous monitoring of digoxin blood concentrations is crucial to ensure optimal therapeutic levels and mitigate the risk of toxicity [23]. While digoxin can serve as a safe and effective treatment for heart failure when employed judiciously, healthcare providers must carefully weigh its potential side effects and interactions [24].

Drug Interactions

Digoxin is susceptible to numerous drug interactions, with 426 drugs identified to interact with it, according to Drugs.com [25]. Significant interactions involve substances such as azole antifungals (itraconazole), dronedarone, macrolide antibiotics (clarithromycin, erythromycin), propafenone, St. John's wort, and certain heart rhythm medications [24,25]. Additionally, digoxin interacts with diuretics, antibiotics, and blood pressure medications [26]. Other interactions encompass substances capable of altering digoxin absorption or excretion, including sucralfate, acarbose, cytotoxic agents, and enzyme inducers [27]. Disease interactions include those related to accessory AV pathways, bradyarrhythmias, AV blocks, hypercalcemia, hypokalemia, hypomagnesemia, preserved left ventricular ejection fraction, renal dysfunction, vasoconstriction, ventricular arrhythmias, acute myocardial infarctions, hyperthyroidism, hypothyroidism, thiamine deficiency, and certain medications taken during pregnancy or while breastfeeding [25]. It is imperative to apprise healthcare providers of all medications being taken, both prescribed and over-the-counter, to minimize potential complications stemming from drug interactions [28].

Challenges and limitations

Narrow Therapeutic Index

Digoxin is renowned for its narrow therapeutic index, indicating that minor fluctuations in plasma concentration can readily result in either toxic or subtherapeutic levels [14]. The generally accepted therapeutic range for digoxin spans from 0.8 to 2.0 ng/mL, with concentrations surpassing 2.4 ng/mL deemed toxic [29]. Nonetheless, discussions have arisen regarding the necessity to revise digoxin's reference ranges owing to disparities in therapeutic thresholds employed by various laboratories [30]. Given its narrow therapeutic window, vigilant monitoring of digoxin levels is imperative, with frequent assessments recommended to uphold concentrations within the optimal range for efficacy and safety [14]. Influential factors, such as age, renal function, and concurrent drug interactions, can significantly influence digoxin pharmacokinetics, underscoring the importance of tailored dosing and meticulous monitoring, particularly in elderly patients [14]. To avert toxicity and ensure favorable therapeutic outcomes, healthcare providers must conscientiously consider comorbidities, drug interactions, electrolyte imbalances, and patient age when prescribing and overseeing digoxin therapy [31]. Consistent serum digoxin level monitoring with thorough clinical evaluation and consideration of pertinent risk factors is indispensable for forestalling adverse effects linked to digoxin utilization [31].

Individual Variability in Response

Individual variability in response to digoxin poses a significant challenge in clinical practice. With its narrow therapeutic index and notable pharmacokinetic variability, achieving appropriate dosing becomes intricate, necessitating meticulous monitoring of serum digoxin concentration levels [32]. Population pharmacokinetic analyses have been instrumental in formulating dosing recommendations for digoxin tailored to specific patient subsets, such as Japanese patients with atrial fibrillation and heart failure [33]. However, despite such efforts, individual factors like age, renal function, and concurrent drug interactions continue to influence digoxin response, mandating personalized dosing strategies and vigilant monitoring [34]. Studies have revealed substantial variations in serum digoxin concentration levels even among patients sharing similar clinical characteristics, underscoring the imperative for meticulous monitoring and individualized therapy management [35,36]. Recognizing individual variability in digoxin response underscores the necessity for a tailored approach to dosing and monitoring, guided by carefully considering patient-specific factors to optimize therapeutic outcomes and minimize the risk of adverse effects.

Emerging Alternatives and Competition

Digoxin, a medication with a rich history in the treatment of heart failure, has encountered competition from newer therapies boasting reduced mortality rates in heart failure patients. Recent trials have failed to definitively establish the mortality benefits of digoxin compared to these innovative treatments, resulting in a decline in its utilization. Nonetheless, upon closer scrutiny of digoxin's pharmacological actions and recent trial findings, it may still hold efficacy in heart failure management [9]. Despite the dwindling use of digoxin, some studies suggest potential advantages, such as diminishing hospital admissions without impacting overall mortality rates [9]. Moreover, individualized dosing strategies and investigations into its effects on vascular smooth muscle cells and neointima formation hint at prospective applications for digoxin in specific patient subsets or conditions [9]. Although alternative therapies have emerged and the utilization of digoxin has waned, ongoing research persists in exploring its potential efficacy and safety profiles, signifying that it may yet retain a role in treating certain cardiovascular conditions. This ongoing exploration underscores the importance of evaluating digoxin's utility and potential benefits in contemporary clinical practice.

Future perspectives

Research Trends and Ongoing Studies

Digoxin, a medication with a longstanding history of use in heart conditions, has recently come under scrutiny regarding its efficacy and safety. Recent studies have presented both positive and negative aspects of digoxin therapy, with some suggesting benefits in specific patient populations while others raise concerns about potential risks [37]. The future perspectives on digoxin therapy entail critically evaluating its role in modern treatment strategies, considering newer alternatives and the evolving landscape of cardiovascular medicine [38]. Research trends and ongoing studies in digoxin focus on comprehending its market dynamics, key drivers, and emerging opportunities. Global digoxin market analysis provides insights into trends embraced by major manufacturers, technological advancements, and competitive landscapes [38]. Despite its historical significance, digoxin's prominence in current practice has diminished due to the availability of safer and more effective therapies for heart failure [38]. In the context of heart failure management, the 2022 AHA/ACC/HFSA guidelines offer updated recommendations that reflect the evolving landscape of cardiovascular care. While digoxin is still mentioned as a potential option in some instances, there needs to be a primary focus in current treatment strategies [21]. The future direction for digoxin therapy lies in continued research to better understand its benefits and risks in modern cardiovascular medicine. This ongoing investigation will be crucial in determining the optimal role of digoxin in contemporary clinical practice.

Potential Novel Applications

The future perspectives on digoxin encompass its potential benefits and challenges as it navigates its role in modern medicine. While historically valuable, ongoing research suggests digoxin may impact autonomic function in patients [37]. However, recent studies have raised safety concerns, associating digoxin treatment with increased mortality and heart failure hospitalizations [39]. In the market landscape, the global digoxin market is undergoing analysis for trends and outlook from 2023 to 2030. Key players like Merck, Pfizer, and Cipla actively engage in this market, prioritizing innovative technologies and sustainable practices [40]. Market segmentation considers factors such as purity levels exceeding or falling below 98% and applications like tablet and injection products [40]. As newer therapies continue to emerge, the future of digoxin may evolve toward more specialized applications or as a secondary treatment option. Understanding the evolving market dynamics and research findings will be pivotal in shaping the future perspectives of digoxin in healthcare, ensuring informed decision-making and optimal patient care.

Technological Advancements in Drug Delivery

Digoxin, a cardiac glycoside utilized in heart failure and specific arrhythmias, boasts a lengthy history of clinical application. However, recent investigations, such as the Digitalis Investigation Group (DIG) trial, have cast doubts on its efficacy and safety, contributing to a decline in its utilization as a first-line treatment [37,41]. Nonetheless, ongoing interest persists in exploring the potential benefits of low-dose digoxin in chronic heart failure, sparking discussions regarding its reconsideration for first-line therapy [41]. Regarding drug delivery technologies, while specific information on technological advancements for digoxin delivery is limited, progress in drug delivery systems at large holds promise for enhancing the efficacy and safety of medications like digoxin. Nanotechnology, targeted drug delivery systems, and controlled-release formulations represent active research areas capable of revolutionizing medication administration, potentially improving patient outcomes, and reducing side effects [42]. Future perspectives on digoxin may encompass novel drug delivery methods to augment its therapeutic effects while mitigating toxicity. These advancements have the potential to reignite interest in digoxin as a viable treatment option for select cardiovascular conditions, laying the groundwork for its resurgence in clinical practice.

Predictions for the Future Role of Digoxin in Clinical Practice

**Figure 1:**
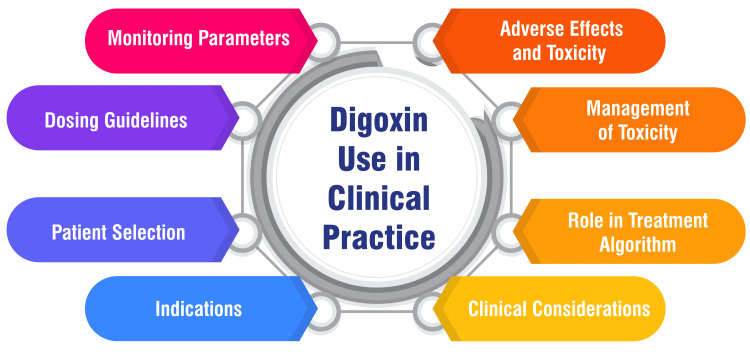
Digoxin use in clinical practice This figure is self-created by the corresponding author.

The future role of digoxin in clinical practice remains to be determined due to conflicting evidence regarding its efficacy and safety. Although historically employed to treat heart conditions, newer therapies boasting improved safety profiles have predominantly supplanted digoxin as first-line treatments. Studies, such as the DIG trial, have failed to demonstrate a clear benefit on overall mortality, prompting scrutiny of its ongoing use [12]. Optimizing the therapeutic benefit of digoxin while mitigating potential harm is paramount, with proposed dosing strategies focusing on achieving low serum levels [43]. The decision to incorporate digoxin into clinical practice should carefully weigh appropriate patient selection against potential adverse effects [37]. Digoxin is often relegated to a secondary treatment option when initial agents prove ineffective [13]. The narrow therapeutic index of digoxin necessitates vigilant monitoring and management to forestall toxicity, which can precipitate serious complications such as arrhythmias and hyperkalemia [20]. Given the dynamic landscape of cardiovascular therapies and the advent of newer drugs, the future role of digoxin may continue to wane in favor of more efficacious and safer treatment alternatives. Further research endeavors and evolving clinical guidelines are poised to delineate the place of digoxin in managing cardiovascular disorders in the years ahead. Digoxin use in clinical practice is shown in Figure 1.

## Conclusions

In conclusion, this review has traversed digoxin's historical trajectory and contemporary relevance, offering a comprehensive examination of its pharmacological properties, clinical applications, efficacy, safety profile, and ongoing challenges. From its origins in traditional medicine to its current status as a cornerstone therapy in cardiovascular medicine, digoxin's journey underscores the intersection of ancient wisdom and modern science. The implications of this review extend beyond the realms of clinical practice to inform ongoing research endeavors, guiding the exploration of digoxin's potential in novel therapeutic domains. Despite the emergence of newer pharmaceuticals, digoxin remains a stalwart in managing heart failure, atrial fibrillation, and other cardiac conditions, owing to its established efficacy, affordability, and global accessibility. As we navigate the complexities of cardiovascular care, digoxin's enduring relevance serves as a poignant reminder of the legacy of medical innovation and the symbiotic relationship between tradition and progress in pursuing optimal patient outcomes.
